# Role of PDK1 in skeletal muscle hypertrophy induced by mechanical load

**DOI:** 10.1038/s41598-021-83098-z

**Published:** 2021-02-10

**Authors:** Naoki Kuramoto, Kazuhiro Nomura, Daisuke Kohno, Tadahiro Kitamura, Gerard Karsenty, Tetsuya Hosooka, Wataru Ogawa

**Affiliations:** 1 Division of Diabetes and Endocrinology, Department of Internal Medicine, Kobe University Graduate School of Medicine, 7-5-1 Kusunoki-cho, Chuo-ku, Kobe, 650-0017 Japan; 2 Metabolic Signal Research Center, Institute for Molecular and Cellular Regulation, Gunma University, Maebashi, Japan; 3 Department of Genetics and Development, Columbia University Irving Medical Center, New York, NY USA; 4 Division of Development of Advanced Therapy for Metabolic Diseases, Department of Internal Medicine, Kobe University Graduate School of Medicine, Kobe, Japan

**Keywords:** Gastroenterology, Molecular medicine

## Abstract

Phosphatidylinositol 3-kinase (PI3K) plays an important role in protein metabolism and cell growth. We here show that mice (M-PDK1KO mice) with skeletal muscle–specific deficiency of 3′-phosphoinositide–dependent kinase 1 (PDK1), a key component of PI3K signaling pathway, manifest a reduced skeletal muscle mass under the static condition as well as impairment of mechanical load–induced muscle hypertrophy. Whereas mechanical load-induced changes in gene expression were not affected, the phosphorylation of ribosomal protein S6 kinase (S6K) and S6 induced by mechanical load was attenuated in skeletal muscle of M-PDK1KO mice, suggesting that PDK1 regulates muscle hypertrophy not through changes in gene expression but through stimulation of kinase cascades such as the S6K-S6 axis, which plays a key role in protein synthesis. Administration of the β_2_-adrenergic receptor (AR) agonist clenbuterol activated the S6K-S6 axis in skeletal muscle and induced muscle hypertrophy in mice. These effects of clenbuterol were attenuated in M-PDK1KO mice, and mechanical load–induced activation of the S6K-S6 axis and muscle hypertrophy were inhibited in mice with skeletal muscle–specific deficiency of β_2_-AR. Our results suggest that PDK1 regulates skeletal muscle mass under the static condition and that it contributes to mechanical load–induced muscle hypertrophy, at least in part by mediating signaling from β_2_-AR.

## Introduction

Not only the function but also the mass of skeletal muscle is an important determinant of whole-body glucose disposal. Low skeletal muscle mass is thus a risk factor for type 2 diabetes (T2D)^1,2^, and sarcopenia, which is characterized by a decline in skeletal muscle mass and consequent impairment of physical activity, is often associated with T2D^3^. Conversely, T2D can trigger a decline in skeletal muscle mass and the development of sarcopenia^4,5^. This bidirectional relation between T2D and muscle mass decline therefore appears to give rise to a vicious cycle.

Insufficient insulin action in skeletal muscle is thought to play an important role in skeletal muscle wasting triggered by T2D^3,6^, although other factors including hyperglycemia and chronic inflammation also likely contribute to this process^3,7,8^. The regulation of skeletal muscle mass depends largely on the balance between the synthesis and degradation of protein^9^, both of which are regulated by insulin^6^. Insulin resistance in humans is associated with impairment of protein anabolism^10^ as well as with muscle mass decline^11,12^, and mice with insulin receptor deficiency specifically in skeletal muscle have a reduced muscle mass^13,14^, indicating the importance of insulin action in the maintenance of skeletal muscle mass.

Phosphatidylinositol 3-kinase (PI3K) plays a key role in various metabolic effects of insulin, including the regulation of protein metabolism^6^. Inhibition of PI3K signaling by genetic ablation of the regulatory subunits of the kinase in mouse skeletal muscle resulted in a loss of skeletal muscle mass^15^, indicating the physiological relevance of such signaling for the regulation of muscle mass. Exercise—in particular, resistance exercise—promotes skeletal muscle hypertrophy. Whereas exercise also appears to stimulate muscle hypertrophy through the regulation of protein metabolism^9,16^, the molecular mechanism underlying the regulation of protein metabolism and skeletal muscle hypertrophy by exercise is not fully understood.

3′-Phosphoinositide–dependent kinase 1 (PDK1) is a serine-threonine protein kinase that phosphorylates and activates downstream kinases such as Akt and ribosomal protein S6 kinase (S6K) in a PI3K-dependent manner^6,17^. It is thus a key element of the PI3K signaling pathway. Whereas most intracellular signaling molecules that mediate insulin action—including insulin receptor substrates, the regulatory and catalytic subunits of PI3K, and the serine-threonine kinases Akt and S6K—have redundant isoforms^6^, PDK1 does not have isoforms that can compensate for its loss of function^6,17^. Ablation of the PDK1 gene thus results in efficient inhibition of insulin action. Mice that lack PDK1 specifically in various organs or cells—including the liver^18,19^, adipocytes^20^, or pancreatic beta cells^21^—have been generated and have revealed corresponding physiological roles of this protein kinase.

We have now generated mice that lack PDK1 specifically in skeletal muscle in order to investigate the role of the muscle enzyme in glucose homeostasis and the regulation of skeletal muscle mass We here show that PDK1 contributes not only to the maintenance of skeletal muscle mass under the static condition but also to the regulation of skeletal muscle hypertrophy induced by mechanical load, at least in part by mediating signaling from the β_2_-adrenergic receptor (β_2_-AR).

## Materials and methods

### Animals

This study was carried out in compliance with the ARRIVE guidelines. All animal experiments were carried out in accordance with the guideline of and approved by the animal experimentation committee of Kobe University Graduate School of Medicine. Mice harboring a floxed allele of the PDK1 gene (PDK1-flox mice)^18^ or of the β_2_-AR gene (*Adrb2*-flox mice)^22^ as well as those expressing Cre recombinase under the control of the myosin light chain 1f. gene promoter (*Mlc1f.*-Cre mice)^23^ were described previously. All mice had been backcrossed to the C57/BL6 background for more than six generations. Mice homozygous for floxed alleles (flox/flox mice) and flox/flox mice harboring the Cre allele (flox/flox-Cre mice) were crossed, and the resultant male flox/flox-Cre (KO) mice and flox/flox (control) mice were studied.

For intraperitoneal glucose tolerance tests, mice deprived of food for 18 h were injected intraperitoneally with glucose (2 g/kg). Blood glucose and plasma insulin concentrations were measured with a glucometer (Sanwa Kagaku Kenkyusho, Nagoya, Japan) and an enzyme-linked immunosorbent assay kit for mouse insulin (Fujifilm Wako Shibayagi, Gunma, Japan), respectively.

### Induction of muscle hypertrophy by synergistic muscle ablation

The gastrocnemius and soleus muscles of both lower limbs were surgical removed from anesthetized mice as previously described^24^. The plantaris muscle was isolated 10 days after surgery and subjected to various assays. As a control, the plantaris muscle of mice that had undergone sham surgery was also collected.

### Cell culture

Mouse C2C12 myoblasts were maintained in growth medium [Dulbecco’s modified Eagle’s medium (DMEM) supplemented with 10% heat-inactivated FBS and 1% penicillin–streptomycin] at 37 °C under a mixture of 95% air and 5% CO_2_ as described previously^8^. Differentiation of the cells was induced by replacement of growth medium with differentiation medium (DMEM supplemented with 2% horse serum and 1% penicillin–streptomycin) at 100% confluence. All experiments with C2C12 myotubes were performed with cells in the fully differentiated state after exposure to differentiation medium for 4 days. Cells were deprived of serum for 15 h before clenbuterol stimulation.

### Adenovirus vector for PDK1 shRNA

An adenovirus vector encoding a short hairpin RNA (shRNA) specific for mouse PDK1 mRNA was generated essentially as described previously^25^. In brief, the nucleotide sequence corresponding to nucleotides 438 to 457 (TGGAGAAACGTCATATTATA) of mouse PDK1 cDNA (GenBank accession number, AF079535) was synthesized by PCR as complementary antiparallel oligomers with a loop sequence^25^ and was then ligated into pcPURmU6icassette (Takara Bio, Ohtsu, Japan), which contains the mouse U6 gene promoter. The resulting construct was ligated into the pAxcwit cosmid cassette (Takara Bio), and adenoviruses encoding PDK1 shRNA (AxshPDK1) were generated with the use of an Adenovirus Expression Vector Kit (Takara Bio). An adenovirus vector containing the U6 promoter alone was used as a control^25^.

### Quantitative RT-PCR analysis, immunoblot analysis, and akt kinase assay

Total RNA was extracted from mouse skeletal muscle with the use of an RNeasy Fibrous Tissue Mini Kit (Qiagen, Hilden, Germany). Quantitative reverse transcription (RT) and PCR analysis was performed as described previously^8^, with 36B4 mRNA as an internal control and with the use of an ABI StepOne Plus Real-Time PCR system (Applied Biosystems, Waltham, MA). The sequences of primer pairs are available on request. For immunoblot analysis, the protein concentration of cell or tissue extracts was measured with a BCA protein assay kit (Thermo Fisher Scientific, Waltham, MA), and the same amount of protein was loaded in each lane. Primary antibodies included those to the following proteins: PDK1 (#3062), Akt (#9272), Thr^308^-phosphorylated Akt (#9275), Ser^473^-phosphorylated Akt (#9271), S6K (#9202), Thr^389^-phosphorylated S6K (#9205), S6 (#2217), Ser^235^- and Ser^236^- phosphorylated S6 (#2211), all from Cell Signaling Technology (Danvers, MA), as well as Thr^229^-phosphorylated S6K (1015B) from R&D Systems (Minneapolis, MN). Akt activity was measured with an Akt Kinase Assay Kit (#9840, Cell Signaling Technology).

### Histological analysis

Skeletal muscle sections were stained with hematoxylin–eosin for quantification of myofiber area with the use of ImageJ software (NIH, Bethesda, MD).

### DNA microarray analysis

Total RNA was extracted from plantaris muscle of wild-type or mutant mice at 10 days after synergistic muscle ablation and was subjected to hybridization with the use of an Affymetrix Mouse Gene 2.0 ST Array (Affymetrix, Santa Clara, CA). Data were analyzed with Transcriptome Viewer (Kurabo, Osaka, Japan). Kyoto Encyclopedia of Genes and Genomes (KEGG) pathway analysis (https://www.kegg.jp/kegg/kegg1.html)^26^ was performed to identify relevant biological pathways with the use of the Database for Annotation, Visualization, and Integrated Discovery (DAVID) v6.7 online tool (http://david.abcc.ncifcrf.gov).

### Indirect calorimetry and locomotor activity measurement

Oxygen consumption and CO_2_ production were measured for individual male mice with an Oxymax apparatus (Columbus Instruments, Columbus, OH). The O_2_ and CO_2_ measurements were performed every 18 min for each mouse over a 3-day period, and the data from the final day were analyzed. Locomotor activity was measured with an ACTIMO-100 system (Shinfactory, Fukuoka, Japan) as described previously^27^.

### Statistical analysis

Quantitative data are presented as means ± SEM. Statistical analysis was performed with Student’s *t* test or one-way ANOVA followed by Tukey’s test, and a *P* value of < 0.05 was considered statistically significant.

### Data and resource availability

Original microarray data were deposited in Gene Expression Omnibus database of the NCBI (GSE150464).

## Results

### Insulin signaling and metabolic phenotype of M-PDK1 KO mice

**Figure 1:**
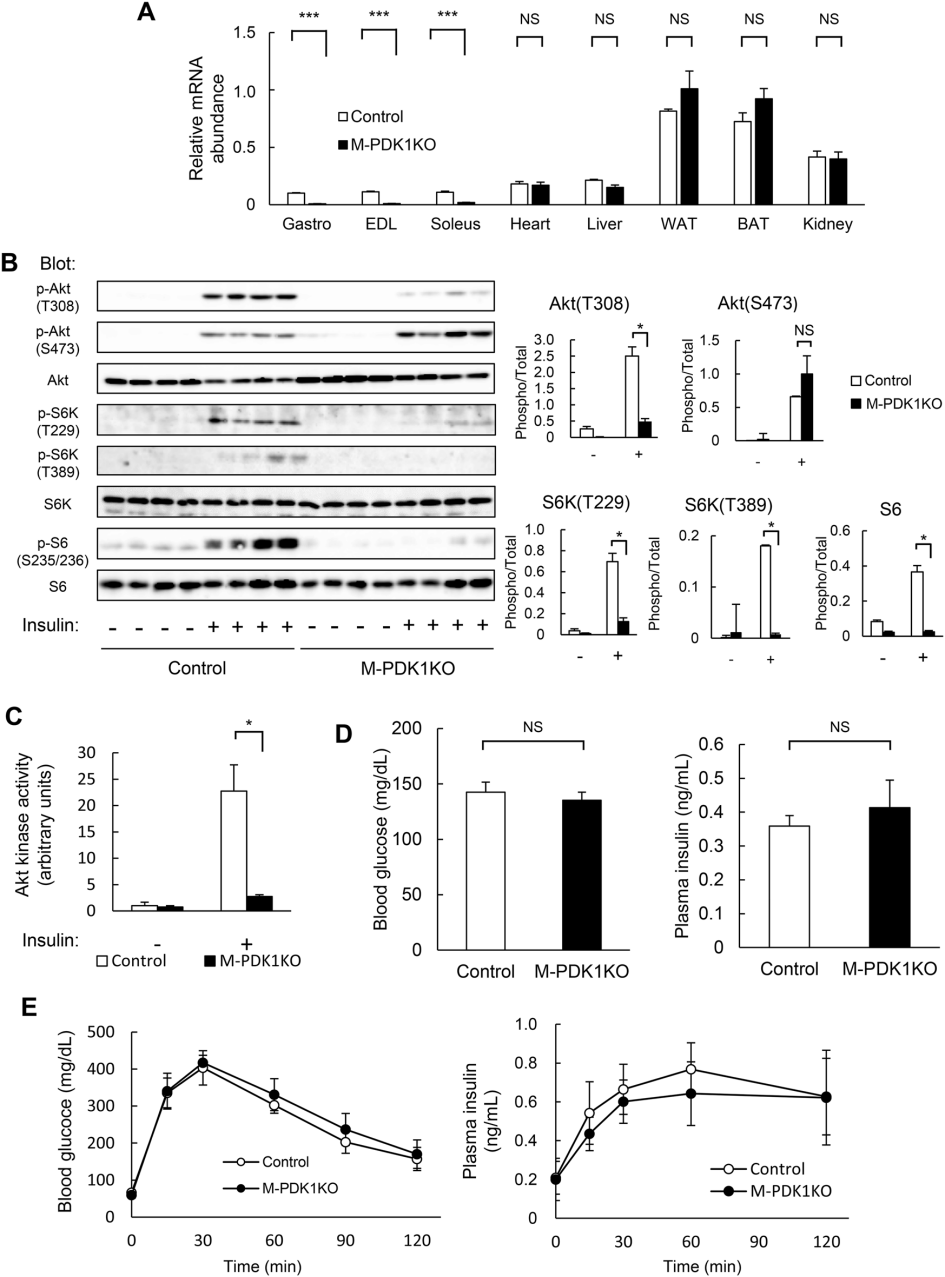
Insulin signaling in skeletal muscle as well as glucose tolerance in M-PDK1KO mice. (**A**) Quantitative RT-PCR analysis of PDK1 mRNA in various tissues of control and M-PDK1KO mice (*n* = 4 each) at 12 weeks of age. Gastro, gastrocnemius; EDL, extensor digitorum longus; WAT and BAT, white and brown adipose tissue, respectively. (**B**,**C**) Immunoblot analysis of insulin signaling molecules (**B**) as well as assay of Akt kinase activity (**C**) in gastrocnemius muscle at 10 min after intraperitoneal injection of human insulin (1 mU/kg) or vehicle in control and M-PDK1KO mice (*n* = 3 each) at 12 weeks of age. In (**B**), each lane of the left panel represents a sample from one mouse, and quantitative data are shown in the left panels. Each band was quantified with the use of the ImageQuant TL software. (**D**,**E**) Blood glucose and plasma insulin levels both in the ad libitum fed state (**D**) and during an intraperitoneal glucose tolerance test (**E**) in control and M-PDK1KO mice [*n* = 9 and 7, respectively, in (**D**); *n* = 6 each in (**E**)] at 12 weeks of age. All quantitative data are means ± SEM. *P < 0.05, ***P < 0.001; NS, not significant [Student’s *t* test in (**A**,**D**,**E**) and one-way ANOVA followed by Tukey’s test in (**B**,**C**)].

We generated mice lacking PDK1 specifically in skeletal muscle (M-PDK1KO mice) by crossing PDK1-flox mice^18^ with *Mlc1f.*-Cre mice^23^. Quantitative RT-PCR analysis confirmed the skeletal muscle–specific ablation of *Pdpk1* in these mice (Fig. 1A). Insulin-induced phosphorylation of Akt at Thr^308^, which is mediated directly by PDK1 and is essential for Akt activity^6,17^, was partially prevented in skeletal muscle of M-PDK1KO mice (Fig. 1B). In contrast, phosphorylation of Akt at Ser^473^ was enhanced in the mutant mice under both basal and insulin-stimulated conditions (Fig. 1B), consistent with the previous finding that loss of PDK1 augments Akt phosphorylation at this site ^19^. Prevention of the insulin-induced activation of Akt in skeletal muscle of M-PDK1KO mice was confirmed with an in vitro assay of kinase activity (Fig. 1C). Insulin-induced phosphorylation of S6K, another protein kinase directly phosphorylated and activated by PDK1^17^, at Thr^229^ and Thr^389^ as well as that of ribosomal protein S6, an endogenous substrate of S6K, were inhibited in skeletal muscle of M-PDK1KO mice (Fig. 1B). Blood glucose and plasma insulin concentrations both in the ad libitum fed state and during a glucose tolerance test were similar in control and M-PDK1KO mice (Fig. 1D,E). Together, these results thus suggested that, whereas insulin-induced activation of Akt and S6K was inhibited, glucose tolerance was maintained in M-PDK1KO mice.

### Reduced skeletal muscle mass in M-PDK1KO mice

**Figure 2:**
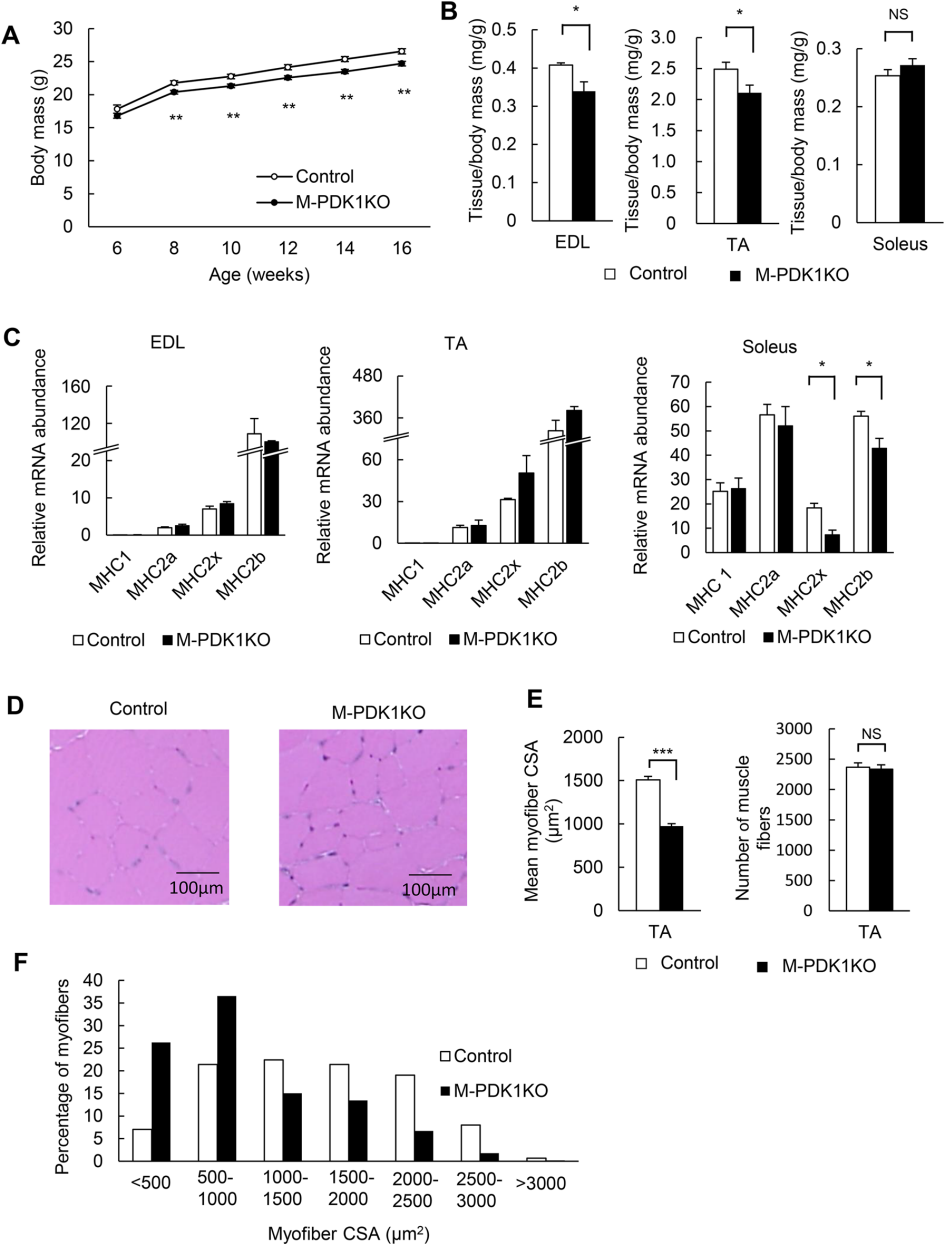
Reduced skeletal muscle mass in M-PDK1KO mice. (**A**,**B**) Body mass at the indicated ages (**A**) and skeletal muscle mass at 12 weeks of age (**B**) for control and M-PDK1KO mice [*n* = 12 and 14, respectively, in (**A**); *n* = 8 each in (**B**)]. (**C**) Quantitative RT-PCR analysis of MHC mRNAs in skeletal muscles of control and M-PDK1KO mice (*n* = 4 each) at 12 weeks of age. (**D–F**) Representative hematoxylin–eosin staining (**D**) as well as the cross-sectional area (CSA) and number of muscle fibers (**E**) and the size distribution of muscle fibers (**F**) in the TA muscle of control and M-PDK1KO mice at 12 weeks of age. For the left panel of (**E**) and for (**F**), 500 fibers from four mice of each genotype were examined; for the right panel of (**E**), the number of fibers in whole cross-sections from four mice of each genotype was counted with the use of ImageJ (https://imagej.nih.gov/ij/). Scale bars in (**D**), 100 μm. Quantitative data are means ± SEM, with the exception of those in (**F**). *P < 0.05, **P < 0.01, ***P < 0.001, NS, not significant [One-way ANOVA followed by Tukey in (**A**) and Student’s *t* test in (**B**,**C**,**E**)].

The body mass of M-PDK1KO mice was slightly but significantly smaller than that of control mice (Fig. 2A). The mass of the extensor digitorum longus (EDL) and tibialis anterior (TA) muscles, in which fast-twitch fibers are predominant, was also smaller in M-PDK1KO mice than in control animals (Fig. 2B), whereas the mass of the soleus muscle, in which slow-twitch fibers are predominant, was similar in the two genotypes (Fig. 2B). The abundance of mRNAs for myosin heavy chains (MHCs) that characterize fast-twitch fibers, such as MHC2b and MHC2x, was reduced in soleus muscle of M-PDK1KO mice (Fig. 2C), whereas that for MHCs characteristic of slow-twitch (MHC1) or fast-twitch (MHC2a, MHC2b and MHC2x) fibers was unaltered in both EDL and TA muscles of the mutant animals (Fig. 2C). These results suggested that the lack of PDK1 impaired the development of fast-twitch fibers and thereby gave rise to a reduced mass of muscles in which fast-twitch fibers are predominant.

Histological analysis revealed that the cross-sectional area of myofibers was decreased, whereas the number of muscles fibers was unaltered, in the TA muscle of M-PDK1KO mice (Fig. 2D,E). The proportions of small and large muscle fibers were increased and decreased, respectively, in TA muscle of M-PDK1KO mice (Fig. 2F), indicating that the smaller skeletal muscle mass of these mice is attributable not to a decrease in the number of muscle fibers but to their atrophy. Oxygen consumption, CO_2_ production, the respiratory exchange ratio (RER), and locomotor activity of M-PDK1KO mice were similar to those of control mice (Supplemental Fig. 1).

### Impaired load-induced muscle hypertrophy in M-PDK1KO mice

**Figure 3:**
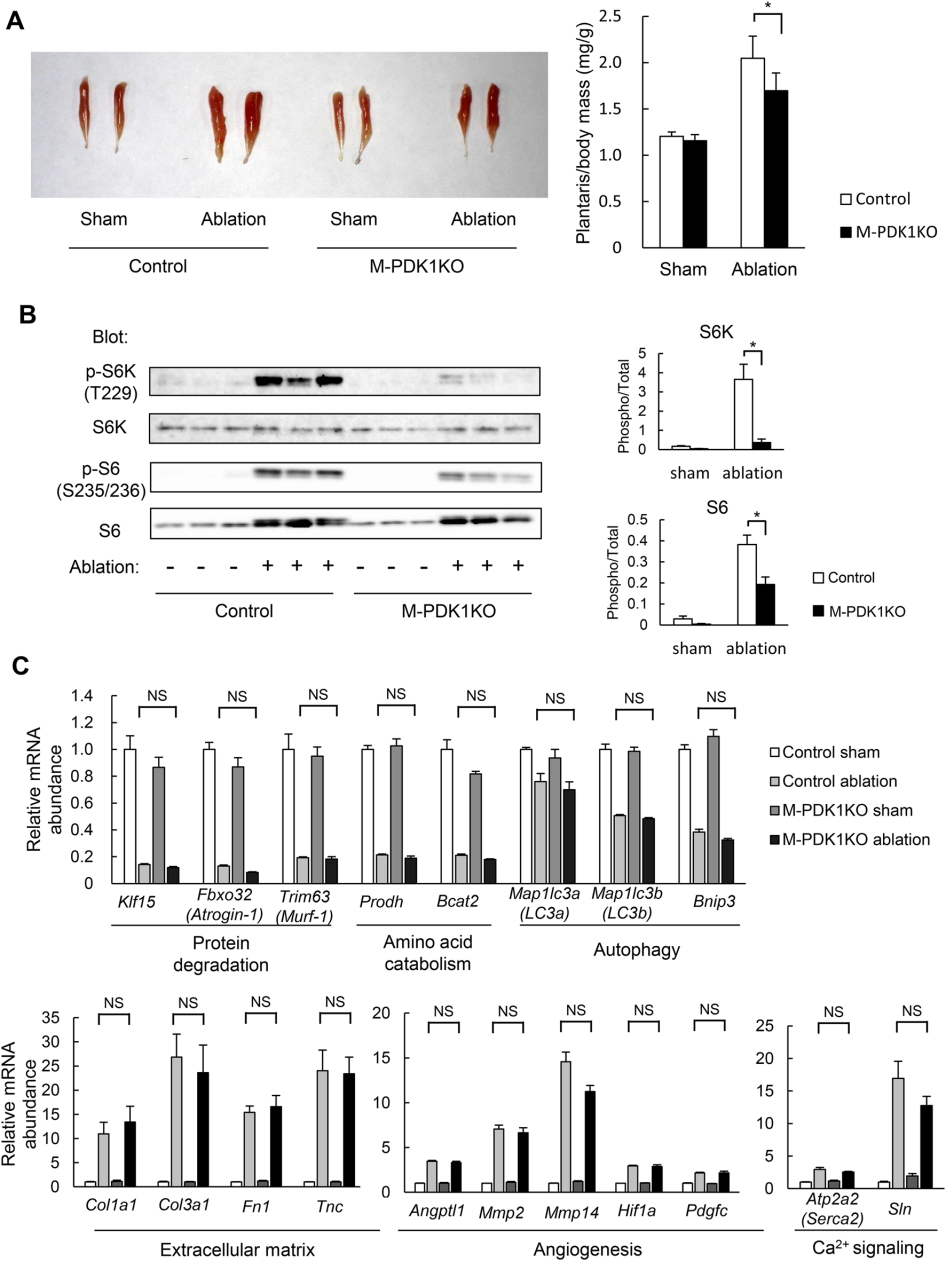
Impairment of exercise-induced muscle hypertrophy in M-PDK1KO mice. (**A**) Representative gross appearance as well as the mass (*n* = 6 mice) of the plantaris muscle at 10 days after synergistic muscle ablation (or sham surgery) in control and M-PDK1KO mice at 12 weeks of age. (**B**) Immunoblot analysis of S6K and S6 abundance and phosphorylation in the plantaris muscle of mice treated as in (**A**). Each lane in the left panel corresponds to one mouse, and quantitative data are shown in the right panels. Each band was quantified with the use of the ImageQuant TL software. (**C**) Quantitative RT-PCR analysis of the indicated mRNAs in the plantaris muscle of mice treated as in (**A**) (*n* = 4 mice). The amount of each mRNA in sham-operated control mice was assigned a value of 1. All quantitative data are means ± SEM. *P < 0.05, NS, not significant [one-way ANOVA followed by Tukey in (**A**,**B**,**C**)].

We next investigated the effect of the lack of PDK1 on muscle hypertrophy triggered by mechanical load. To this end, we studied mice with synergistic muscle ablation. Surgical muscle ablation results in hypertrophy of muscles that function synergistically with the ablated muscles as a result of compensatory exercise overload^24^. In control animals, ablation of the gastrocnemius and soleus muscles resulted in an ~ 1.5-fold increase in the mass of the plantaris muscle, which contributes to flexion of the lower limbs in coordination with the gastrocnemius and soleus, within 10 days (Fig. 3A). Such hypertrophy of the plantaris muscle induced by synergistic muscle ablation was inhibited by ~ 30% in M-PDK1KO mice (Fig. 3A). Ablation of the gastrocnemius and soleus increased the abundance as well as the phosphorylation of both S6K and S6, markers of protein synthesis, in the plantaris muscle of control mice (Fig. 3B). Phosphorylation of these proteins in response to synergistic muscle ablation was markedly inhibited in M-PDK1KO mice (Fig. 3B).

Given that skeletal muscle mass is determined by a balance between the synthesis and degradation of muscle proteins^15,16^, we next investigated the expression of genes related to protein degradation in mice subjected to synergistic muscle ablation. The abundance of mRNAs derived from genes related to general protein degradation (*Klf15*, *Fbxo32*, and *Trim36*), amino acid catabolism (*Prodh* and *Bcat2*), and autophagy (*Map1lc3a*, *Map1lc3b* and *Bnip3*) was down-regulated in the hypertrophic plantaris muscle of control animals (Fig. 3C), indicating that protein degradation may be suppressed at the level of gene expression during muscle growth induced by synergistic muscle ablation. The abundance of all these mRNAs was similarly down-regulated in the plantaris muscle of M-PDK1KO mice in response to synergistic muscle ablation (Fig. 3C).

In contrast, the expression of genes related to extracellular matrix formation (*Col1a1*, *Col3a1*, *Fn1*, and *Tnc*) was up-regulated in the hypertrophic plantaris muscle of control mice (Fig. 3C), consistent with previous observations^28^. The expression of genes related to angiogenesis (*Angptl1*, *Mmp2*, *Mmp14*, *HIF1a*, and *Ptgfc*) or to cellular Ca^2+^ signaling (*Atp2a2* and *Sln*) was also up-regulated in the control mice (Fig. 3C). The up-regulation of all these genes was similarly apparent in the hypertrophic plantaris muscle of M-PDK1KO mice (Fig. 3C).

We further analyzed changes in the expression of genes during the development of muscle hypertrophy induced by synergistic muscle ablation with the use of a DNA microarray. Such analysis revealed that the expression of various categories of genes was altered in response to synergetic muscle ablation (Supplemental Fig. 2). Categories of genes whose expression was up-regulated in the hypertrophic plantaris muscle of control animals included PI3K-Akt signaling, focal adhesion, pathways in cancer, phagosome, and proteoglycans in cancer, whereas those whose expression was down-regulated included metabolic pathways, biosynthesis of antibiotics, calcium signaling pathway, and insulin signaling pathway (Supplemental Fig. 2C,E). Similar changes in gene expression were apparent in M-PDK1KO mice (Supplemental Fig. 2D,F).

Together, these results thus suggested that PDK1 plays an important role in mechanical load–induced muscle hypertrophy, but that this function of the kinase is not mediated by changes in gene expression.

### Role of β_2_-adrenergic signaling in skeletal muscle hypertrophy

**Figure 4:**
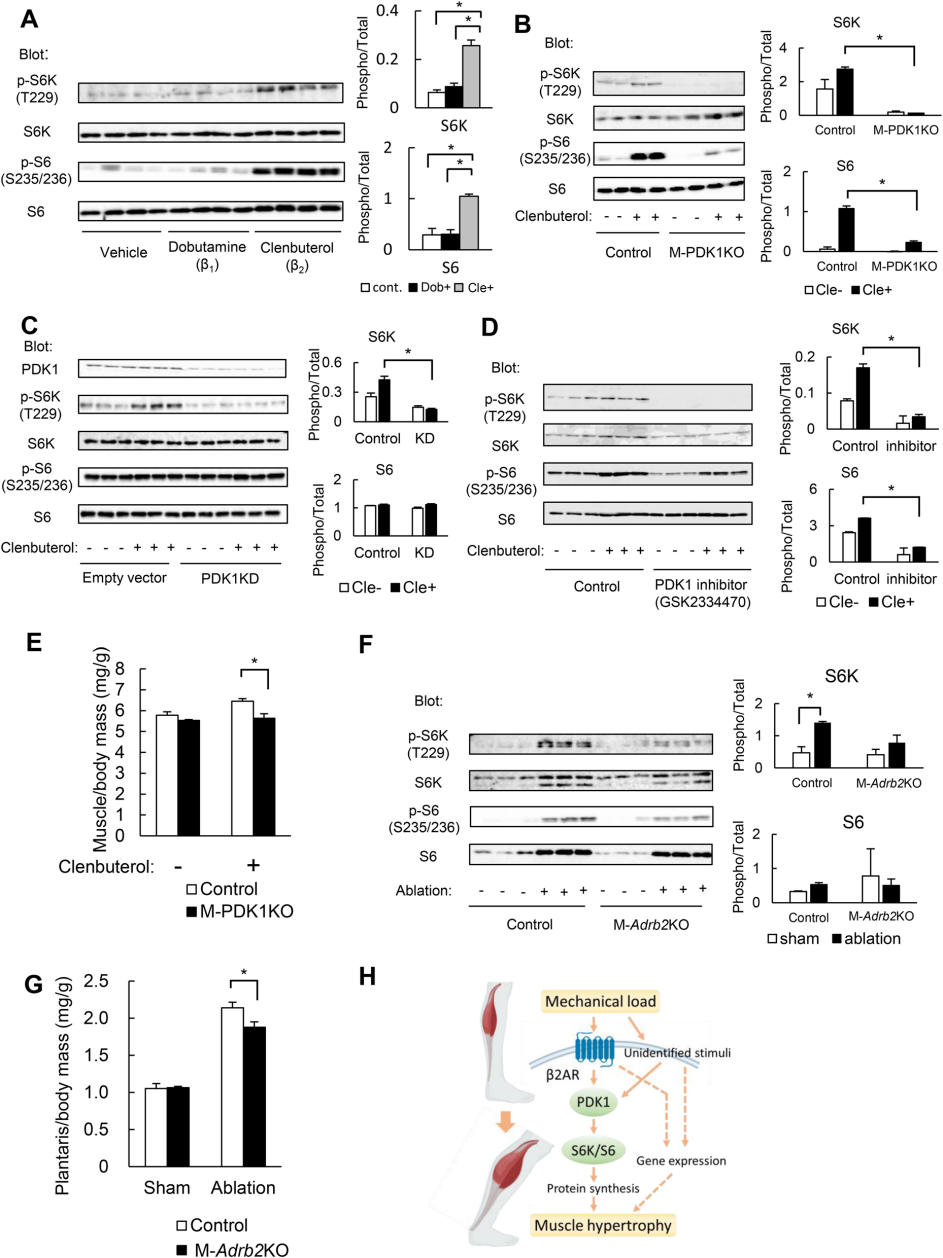
Role of β_2_-AR signaling in exercise-induced skeletal muscle hypertrophy. (**A**,**B**)*:* Immunoblot analysis of the abundance and phosphorylation of S6K and S6 in gastrocnemius muscle of control mice (**A**) or of control and M-PDK1KO mice (**B**) at 12 weeks of age and at 10 min after intraperitoneal injection of clenbuterol (1 mg/kg) (**A**,**B**), dobutamine (1 mg/kg) (**A**), or vehicle. (**C**,**D**) Immunoblot analysis of S6K and S6 abundance and phosphorylation in serum-deprived C2C12 myotubes infected with an adenovirus encoding PDK1 shRNA (PDK1KD) or with a control virus (empty vector) (**C**) or incubated with a pharmacological inhibitor (GSK2334470, 3 μM) for 30 min (**D**),and then incubated in the absence or presence of 100 nM clenbuterol for 10 min. (**E**) Mass of the gastrocnemius muscle 10 days after daily intraperitoneal injection of clenbuterol (1 mg/kg) or vehicle in control and M-PDK1KO mice (*n* = 4 each) at 12 weeks of age. (**F**) Immunoblot analysis of S6K and S6 abundance and phosphorylation in the plantaris muscle at 10 days after synergistic muscle ablation (or sham surgery) in control and M-*Adrb2*KO mice at 12 weeks of age. (**G**) Mass of the plantaris muscle for mice as in (**F**) (*n* = 4 and 5 mice of each genotype for sham surgery and muscle ablation, respectively). Each lane in (**A**,**B**,**F**) represents a sample from one mouse; each lane in (**C**,**D**) represents one dish, and quantitative data are shown in the right panels. Each band was quantified with the use of the ImageQuant TL software. All quantitative data are means ± SEM. *P < 0.05, NS, not significant (one-way ANOVA followed by Tukey). (**H**) Proposed role for the β_2_-AR–PDK1 signaling pathway in mechanical load induced muscle hypertrophy. Whereas PDK1 and β_2_-AR contribute to load–induced skeletal muscle hypertrophy, and that PDK1 mediates, at least in part, the effect of β_2_-AR signaling, unidentified stimuli may also contribute to load-induced muscle hypertrophy upstream of PDK1. Alterations in gene expression, which are independent of PDK1, may be attributable to the muscle hypertrophic effect. Dashed lines represent unidentified signaling pathways.

Finally, we investigated extracellular stimuli that might be responsible for the induction of muscle hypertrophy by mechanical load. We focused on the possible role of β-AR signaling, which is known to contribute to skeletal muscle hypertrophy^29^. Administration of the β_2_-AR agonist clenbuterol, but not that of the β_1_-AR agonist dobutamine, was found to stimulate phosphorylation of S6K and S6 in skeletal muscle (Fig. 4A). These effects of clenbuterol were markedly attenuated in M-PDK1KO mice (Fig. 4B), indicating that PDK1 is essential for the activation of S6K-S6 signaling in response to β_2_-AR stimulation. Furthermore, treatment of C2C12 myotubes with clenbuterol resulted in an increase in the phosphorylation of S6K, and this effect was inhibited by shRNA-mediated depletion of (Fig. 4C) or by a pharmacological inhibitor for PDK1 (Fig. 4D), confirming the important role of PDK1 in β_2_-AR signaling.

We further analyzed the role of the β_2_-AR–PDK1 axis in muscle hypertrophy in mice. Intraperitoneal administration of clenbuterol for 10 days resulted in an ~ 7% increase in gastrocnemius muscle mass in control mice, whereas it had no such effect in M-PDK1KO mice (Fig. 4E). To confirm the physiological relevance of β_2_-AR signaling in skeletal muscle hypertrophy, we generated mice that lack β_2_-AR specifically in skeletal muscle (M-*Adrb2*KO mice) by crossing *Adrb2*-flox mice^22^ and *Mlc1f.*-Cre mice^23^. Body and skeletal muscle mass as well as blood glucose and plasma insulin levels in the ad libitum fed state did not differ significantly between M-*Adrb2*KO and control mice at 12 weeks of age (data not shown). The increases in the abundance and phosphorylation of S6K and S6 induced in plantaris muscle by synergistic muscle ablation were attenuated in M-*Adrb2*KO mice (Fig. 4F). Whereas synergistic muscle ablation induced an ~ twofold increase in plantaris muscle mass in control animals, this effect was inhibited by ~ 25% in M-*Adrb2*KO mice (Fig. 4G). These results suggested that the β_2_-AR–PDK1 signaling pathway plays an important role in mechanical load–induced skeletal muscle hypertrophy. Ccomplete unedited blots are shown in Supplemental Fig. 3.

## Discussion

We have here shown that mice with skeletal muscle–specific deficiency of PDK1, a key participant in the PI3K signaling pathway^17^, manifest a reduced skeletal muscle mass under the static condition as well as impairment of the induction of muscle hypertrophy in response to mechanical load. Given that PI3K plays a key role in cell growth and protein synthesis induced by hormones and growth factors including insulin, the PI3K signaling pathway has been thought to contribute to exercise-induced muscle hypertrophy^16^. We have now provided the first genetic evidence that this pathway plays an essential role in muscle hypertrophy induced by mechanical load.

We also found that PDK1 plays an important role in muscle hypertrophy induced by β_2_-AR stimulation, and that mice with skeletal muscle–specific deficiency of β_2_-AR also manifest impairment of exercise-induced muscle hypertrophy. β_2_-AR agonists are known to increase skeletal muscle mass in both animals and humans^29,30^ and have been administered as doping agents by athletes^31^. Our results have now uncovered the physiological relevance of β_2_-AR signaling and the importance of PDK1 as a downstream signaling molecule in muscle hypertrophy induced by mechanical load. The inhibition of mechanical load–induced muscle hypertrophy appeared to be greater in M-PDK1KO mice than in M-*Adrb2*KO mice, suggesting that signals other than that from β_2_-AR might contribute to exercise-induced muscle hypertrophy upstream of PDK1.

Mice with skeletal muscle–specific deficiency of the insulin receptor show a mild reduction in skeletal muscle mass, whereas skeletal muscle mass was found to be unaltered in mice with skeletal muscle–specific deficiency of the insulin growth factor–1 (IGF-1) receptor^13,14^. Moreover, mice lacking both of these receptors in skeletal muscle manifested a marked reduction of skeletal muscle mass^13,14^. These observations suggest that the role of the insulin receptor in maintenance of muscle mass is predominant, and that the IGF-1 receptor compensates for loss of signaling from the insulin receptor. Given that the extent of the reduction in skeletal muscle mass apparent in M-PDK1KO mice was similar to that in mice with muscle-specific deficiency of the insulin receptor rather than to that in mice with muscle-specific deficiency of both the insulin and IGF-1 receptors, the compensatory effect of the IGF-1 receptor is likely mediated via a pathway independent of PDK1 signaling. Mice in which PI3K signaling was ablated in skeletal muscle also showed a relatively mild reduction in skeletal muscle mass similar to that observed in M-PDK1KO mice^15^.

The reduction in muscle mass induced by the loss of both the insulin and IGF-1 receptors was almost completely prevented by additional disruption of FoxO-family transcription factors^14^, indicating that the regulation of muscle mass by these receptors under the static condition is largely dependent on transcriptional regulation. We have now shown that the expression of various categories of genes was altered during the induction of muscle hypertrophy by synergistic muscle ablation, and these changes in gene expression were similarly observed in M-PDK1KO mice. Our results suggest that PDK1 contributes to mechanical load–induced muscle hypertrophy not through the regulation of gene expression given that mechanical load–induced alterations in gene expression was not prevented by the lack of PDK1. It is likely that protein kinase cascades activated by PDK1, such as the S6K-S6 axis, might contribute to this process. Nevertheless, the marked changes in gene expression likely contribute to muscle hypertrophy, with the residual muscle hypertrophy apparent in M-PDK1KO mice likely being attributable to these gene expression changes remaining intact. It will thus be important to elucidate the mechanism by which exercise-induced gene expression in skeletal muscle is regulated.

We found that glucose tolerance was not impaired in M-PDK1KO mice. Mice with skeletal muscle–specific deficiency of the insulin receptor also show normal glucose tolerance^32^. These observations indicate that glucose tolerance is well compensated in mice in which insulin signaling in skeletal muscle is inhibited either at the level of the receptor or of PDK1. However, glucose intolerance was evident in mice in which PI3K signaling in skeletal muscle was inhibited by muscle-specific disruption of *Pik3r1* (which encodes the p85α, p55α, and p50α regulatory subunits of PI3K) together with whole-body disruption of *Pik3r2* (which encodes the p85β regulatory subunit)^15^. The reason for this apparent discrepancy is unclear, but it is possible that the lack of p85β in the whole body might have some influence.

In conclusion, we have shown that PDK1 and β_2_-AR contribute to mechanical load–induced skeletal muscle hypertrophy, and that PDK1 mediates, at least in part, the hypertrophic effect of β_2_-AR signaling (Fig. 4H). The potential clinical application of β_2_-AR agonists to muscle-wasting diseases has attracted much attention. As a means of minimizing adverse effects of such drugs on the cardiovascular system, tissue-selective agonists are also under development^33^. Further analysis of the role of the β_2_-AR–PDK1 signaling pathway in muscle hypertrophy will likely contribute to the development of novel drugs for muscle-wasting diseases.

## Supplementary Information

Supplementary Information 1.
