# Supplementary material for: Role of PDK1 in skeletal muscle hypertrophy induced by mechanical load
Source: Sci Rep. 2021 Feb 10;11:3447. doi: 10.1038/s41598-021-83098-z (PMC7876046; doi:10.1038/s41598-021-83098-z)
Supplement: Supplementary file 1 — Supplementary Information 1. [file 41598_2021_83098_MOESM1_ESM.pdf]

Supplemental Information

Role of PDK1 in Skeletal Muscle Hypertrophy Induced by Mechanical Load

Naoki Kuramoto, Kazuhiro Nomura, Daisuke Kohno, Tadahiro Kitamura, Gerard Karsenty, Tetsuya Hosooka, and Wataru Ogawa

Supplemental Fig. 1—Energy consumption and locomotor activity of M-PDK1KO mice. VO_2 (*A*), VCO_2 (*B*), the respiratory exchange ratio (*C*), and locomotor activity (*D*) were measured with metabolic cages for control and M-PDK1KO mice at 12 weeks of age (n = 4 each). Measured values every 18 min are shown on the left, and average values for the light and dark phases are shown on the right. Data are means ± SEM. NS, not significant (Student's *t* test).

Supplementary Fig. 2—DNA microarray analysis of gene expression in plantaris muscle after synergistic muscle ablation. *A* and *B:* Heat maps for up-regulated genes (*A*) and down-regulated genes (*B*) in plantaris muscle of control and M-PDK1KO mice at 10 days after synergistic muscle ablation. Up-regulated and down-regulated genes were defined with cutoffs of 2- and 0.5-fold changes, respectively, relative to expression levels in corresponding sham-operated mice. *C–F:* The top eight KEGG pathways for up-regulated genes in control mice (*C*) or M-PDK1KO mice (*D*) as well as for down-regulated genes in control mice (*E*) or M-PDK1KO mice (*F*). *$P < 0.05$, **$P < 0.01$, ***$P < 0.001$ (Student's *t* test).

Supplementary Fig. 3— Uncropped blot images. Black box indicates area that was cropped and displayed in the indicated.

Supplementary Figure 1

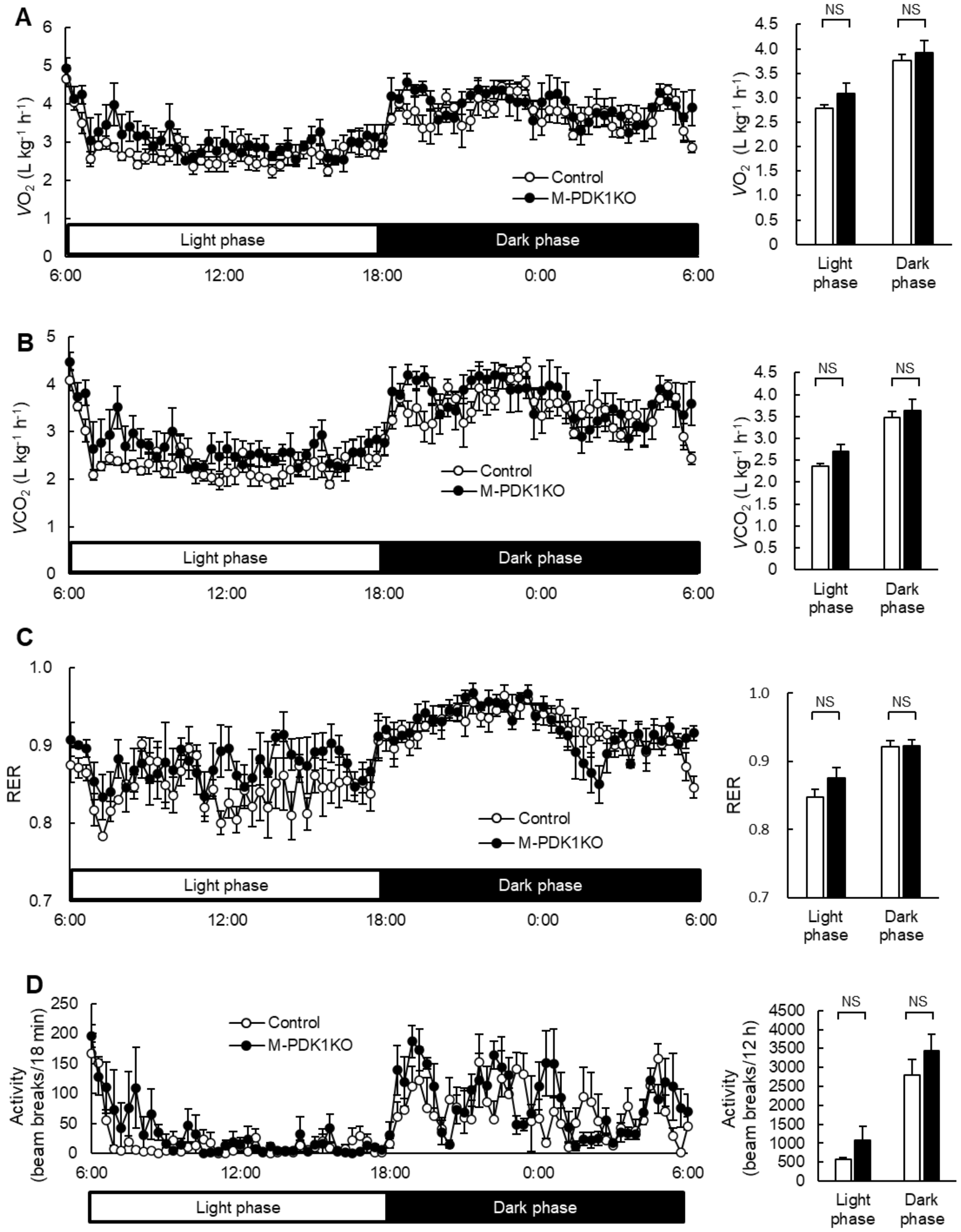

Supplementary Figure 2

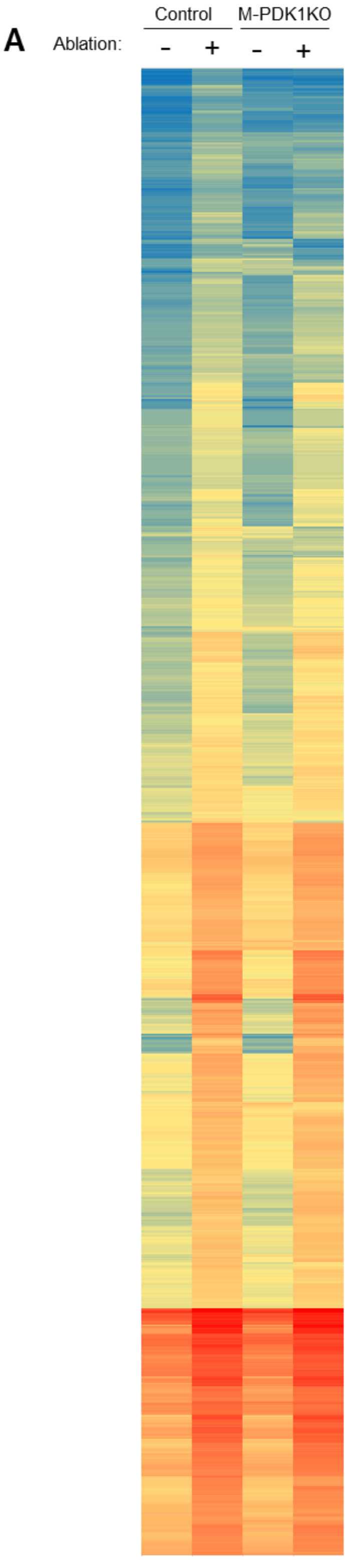

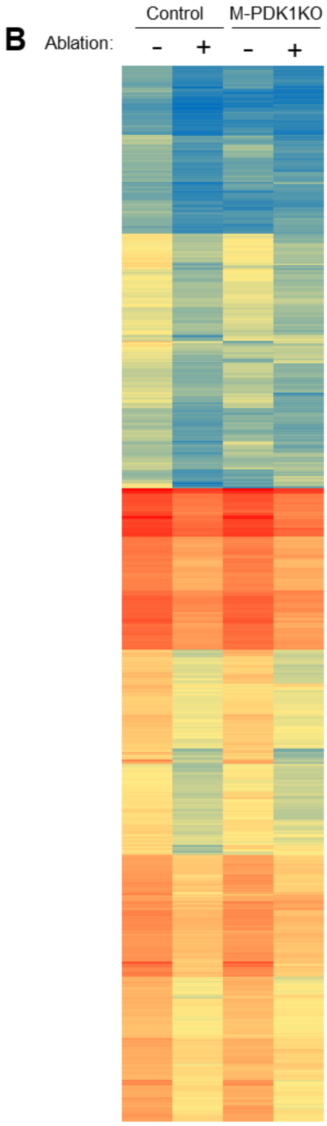

C

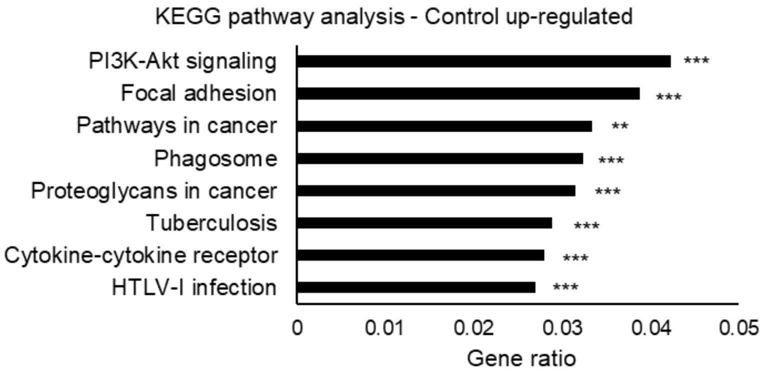
KEGG pathway analysis - Control up-regulated
PI3K-Akt signaling ***
Focal adhesion ***
Pathways in cancer **
Phagosome ***
Proteoglycans in cancer ***
Tuberculosis ***
Cytokine-cytokine receptor ***
HTLV-I infection ***
0
0.01
0.02
0.03
0.04
0.05
Gene ratio

D

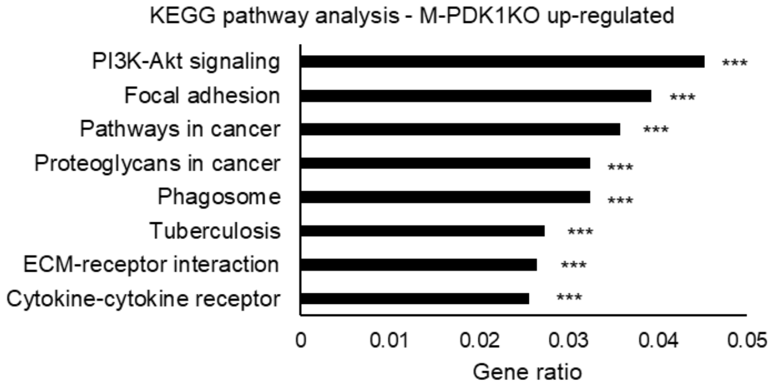
KEGG pathway analysis - M-PDK1KO up-regulated
PI3K-Akt signaling ***
Focal adhesion ***
Pathways in cancer ***
Proteoglycans in cancer ***
Phagosome ***
Tuberculosis ***
ECM-receptor interaction ***
Cytokine-cytokine receptor ***
0
0.01
0.02
0.03
0.04
0.05
Gene ratio

E

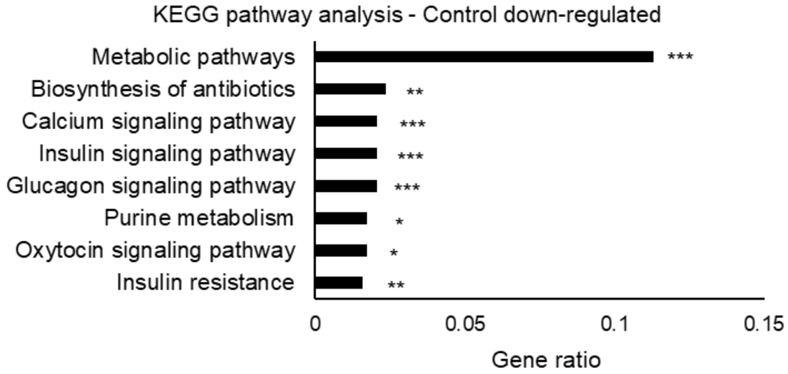
KEGG pathway analysis - Control down-regulated
Metabolic pathways ***
Biosynthesis of antibiotics **
Calcium signaling pathway ***
Insulin signaling pathway ***
Glucagon signaling pathway ***
Purine metabolism *
Oxytocin signaling pathway *
Insulin resistance **
0
0.05
0.1
0.15
Gene ratio

F

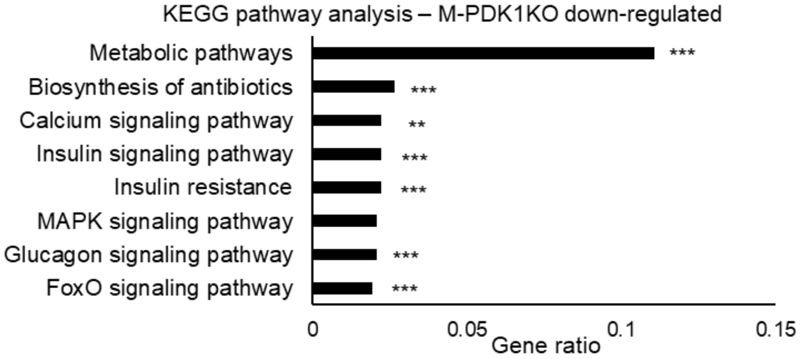
KEGG pathway analysis – M-PDK1KO down-regulated
Metabolic pathways ***
Biosynthesis of antibiotics ***
Calcium signaling pathway **
Insulin signaling pathway ***
Insulin resistance ***
MAPK signaling pathway
Glucagon signaling pathway ***
FoxO signaling pathway ***
0
0.05
0.1
0.15
Gene ratio

Supplementary Figure 3

Figure 1B

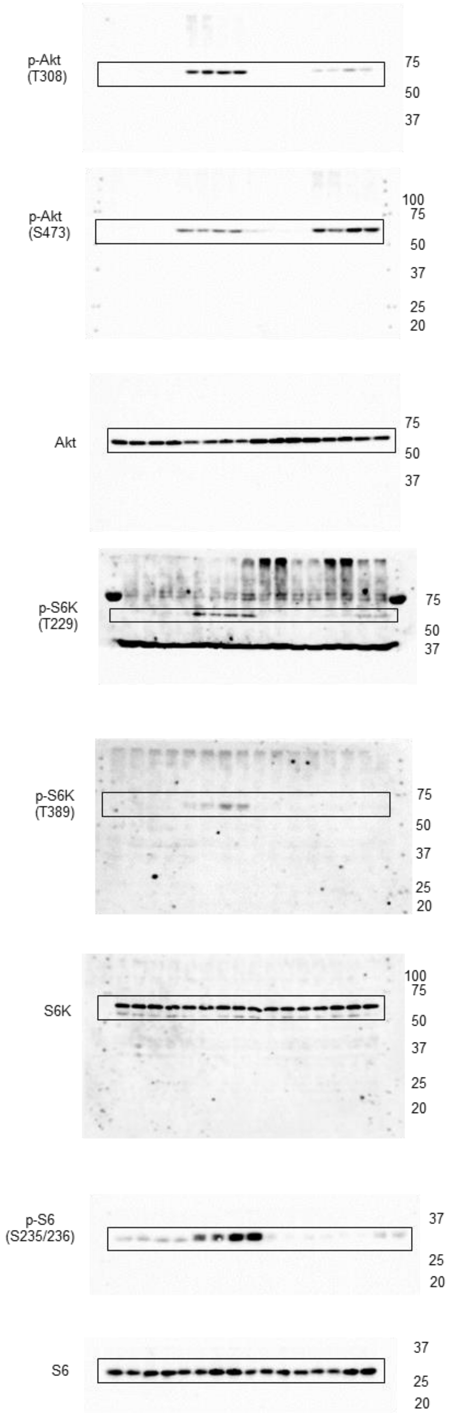

Figure 3B

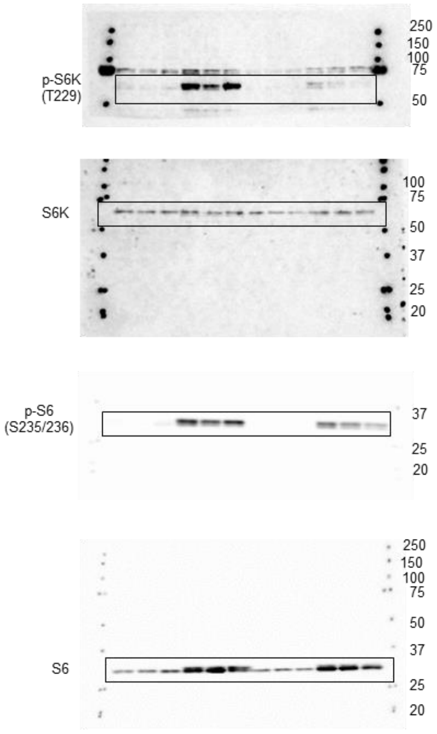

Figure 4A

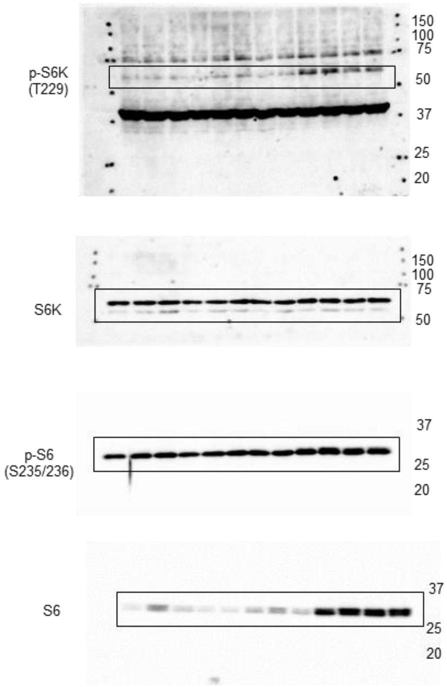

Figure 4B

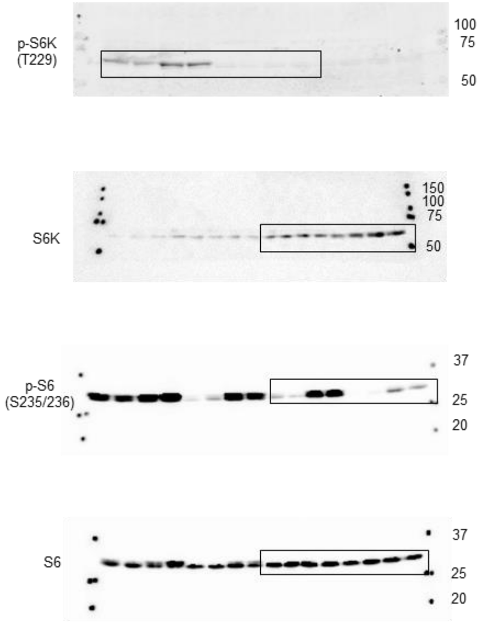

Figure 4C

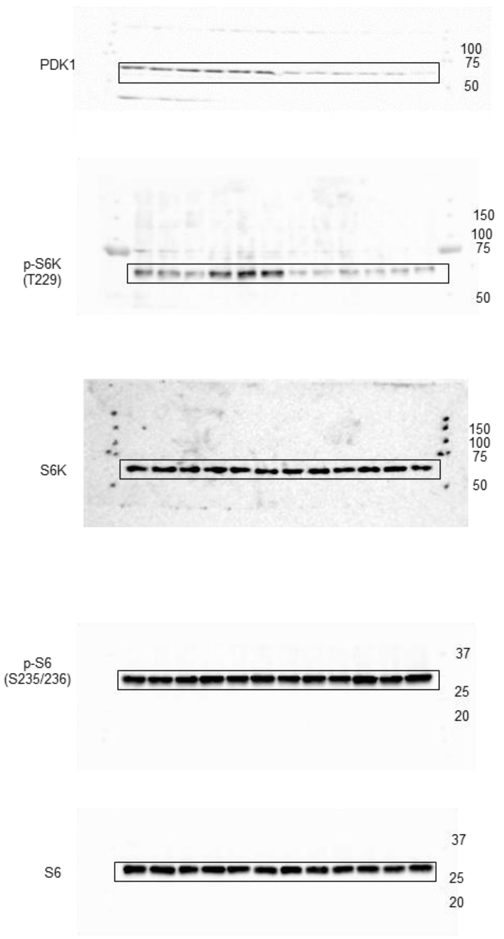

Figure 4D

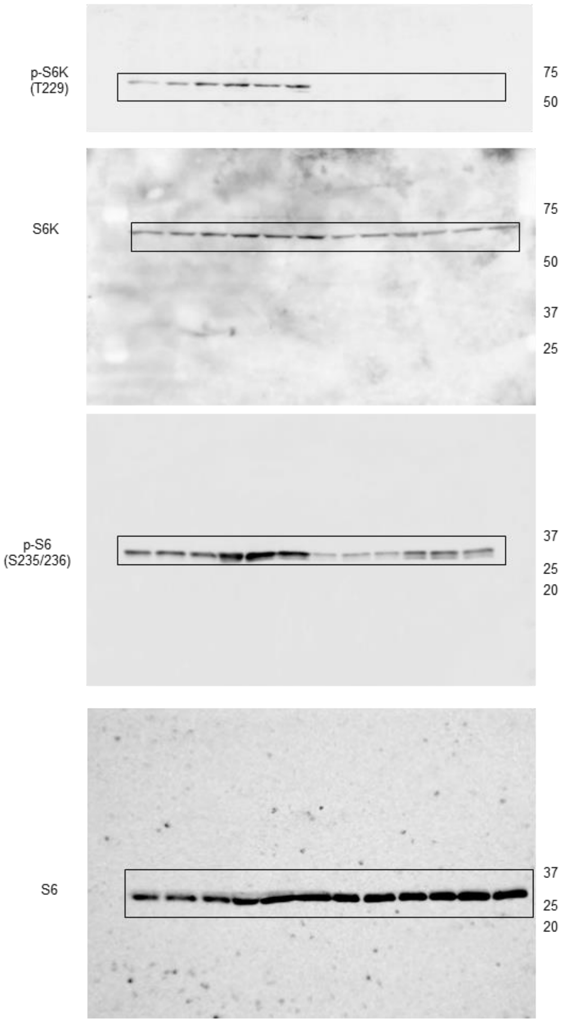

Figure 4F

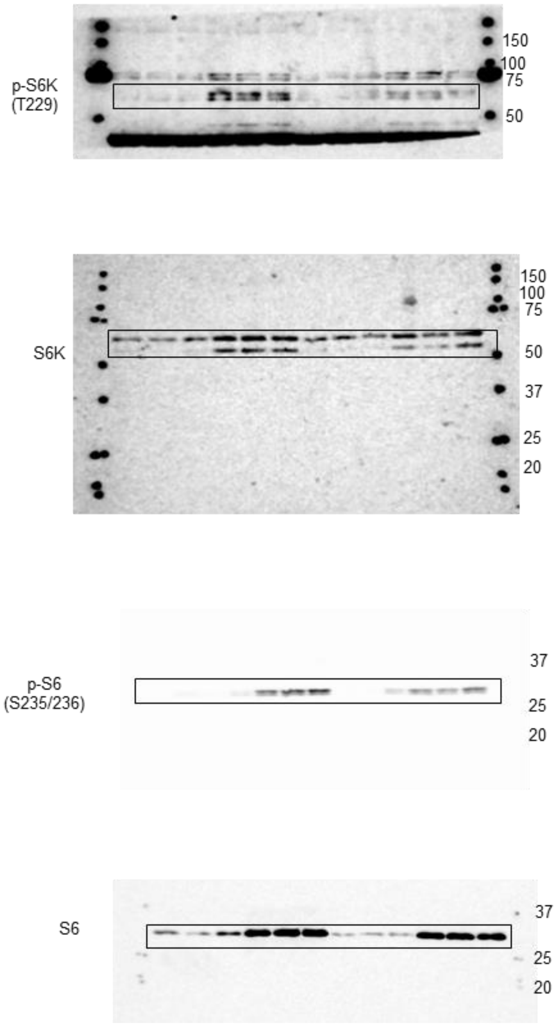

p-S6K
(T229)
150
100
75
50
S6K
150
100
75
50
37
25
20
p-S6
(S235/236)
37
25
20
S6
37
25
20
